# 5-Chloro­benzothia­zole-2-spiro-3′-indolin-2′-one

**DOI:** 10.1107/S1600536810001285

**Published:** 2010-01-20

**Authors:** Mehmet Akkurt, Selvi Karaca, Görkem Ermut, Nilgün Karalı, Orhan Büyükgüngör

**Affiliations:** a Department of Physics, Faculty of Arts and Sciences, Erciyes University, 38039 Kayseri, Turkey; b Department of Pharmaceutical Chemistry, Faculty of Pharmacy, stanbul University, 34116 Beyazıt–stanbul, Turkey; c Department of Physics, Faculty of Arts and Sciences, Ondokuz Mayıs University, 55139 Samsun, Turkey

## Abstract

The title compound, C_14_H_9_ClN_2_OS, crystallizes with two unique mol­ecules, *A* and *B*, in the asymmetric unit. The five-membered rings of the benzothia­zole groups in both mol­ecules adopt an envelope conformation [puckering parameters: *q*_2_ = 0.242 (1) Å and ϕ_2_ = 217.5 (4)° for *A*, and *q*_2_ = 0.234 (1) Å and ϕ_2_ = 37.7 (4)° for *B*]. The five-membered rings of the indolinone groups in both mol­ecules are also not planar, with a twisted conformation [puckering parameters are *q*_2_ = 0.112 (2) Å and ϕ_2_ = 126.3 (8)° for *A*, and *q*_2_ = 0.108 (2) Å and ϕ_2_ = 306.4 (9)° for *B*]. In the crystal structure, there are inter­molecular N—H⋯O, N—H⋯S and C—H⋯O hydrogen-bonding inter­actions, forming the layers propagating normal to *c*.

## Related literature

For general background to and applications of 1*H*-indole-2,3-dione derivatives, see: Alam & Nawwar (2002 ▶); Cho *et al.* (2008 ▶); Da-Silva *et al.* (2001 ▶); Dandia *et al.* (1990 ▶); Hall *et al.* (2009 ▶); Joshi *et al.* (1990 ▶); Kumar *et al.* (2008 ▶); Quenelle *et al.* (2006 ▶); Vine *et al.* (2007 ▶, 2009 ▶); Ćaleta *et al.* (2009 ▶). For bond-length data, see: Allen *et al.* (1987 ▶). For puckering parameters, see: Cremer & Pople (1975 ▶).
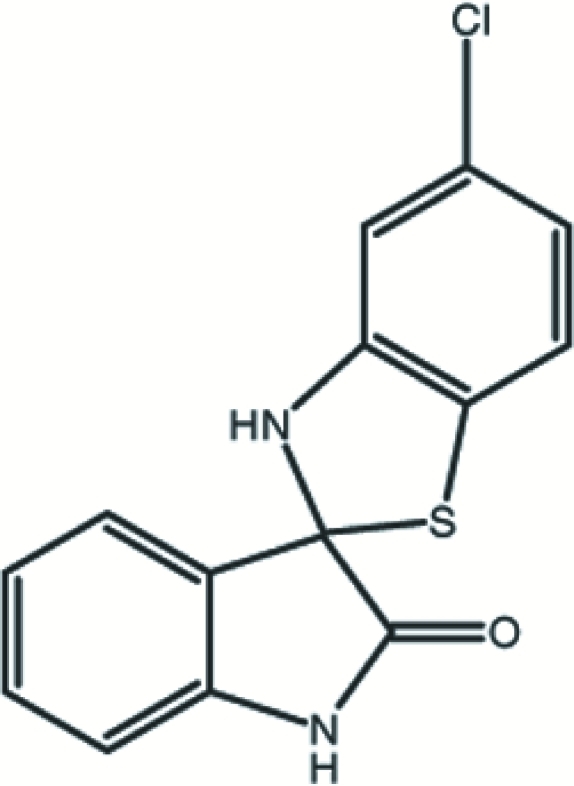


## Experimental

### 

#### Crystal data

C_14_H_9_ClN_2_OS
*M* *_r_* = 288.75
Monoclinic, 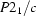

*a* = 12.8421 (6) Å
*b* = 9.1159 (3) Å
*c* = 22.1553 (9) Å
β = 97.051 (3)°
*V* = 2574.1 (2) Å^3^
*Z* = 8
Mo *K*α radiation
μ = 0.45 mm^−1^
*T* = 295 K
0.77 × 0.49 × 0.19 mm

#### Data collection

Stoe IPDS 2 diffractometer
Absorption correction: integration (*X-RED32*; Stoe & Cie, 2002 ▶) *T*_min_ = 0.723, *T*_max_ = 0.919
23976 measured reflections
5271 independent reflections
3987 reflections with *I* > 2σ(*I*)
*R*_int_ = 0.031

#### Refinement

*R*[*F* ^2^ > 2σ(*F* ^2^)] = 0.033
*wR*(*F* ^2^) = 0.080
*S* = 1.01
5271 reflections
359 parameters
H atoms treated by a mixture of independent and constrained refinement
Δρ_max_ = 0.17 e Å^−3^
Δρ_min_ = −0.19 e Å^−3^

### 

Data collection: *X-AREA* (Stoe & Cie, 2002 ▶); cell refinement: *X-AREA*; data reduction: *X-RED32* (Stoe & Cie, 2002 ▶); program(s) used to solve structure: *SIR97* (Altomare *et al.*, 1999 ▶); program(s) used to refine structure: *SHELXL97* (Sheldrick, 2008 ▶); molecular graphics: *ORTEP-3 for Windows* (Farrugia, 1997 ▶); software used to prepare material for publication: *WinGX* (Farrugia, 1999 ▶).

## Supplementary Material

Crystal structure: contains datablocks global, I. DOI: 10.1107/S1600536810001285/im2170sup1.cif

Structure factors: contains datablocks I. DOI: 10.1107/S1600536810001285/im2170Isup2.hkl

Additional supplementary materials:  crystallographic information; 3D view; checkCIF report

## Figures and Tables

**Table 1:** Hydrogen-bond geometry (Å, °)

*D*—H⋯*A*	*D*—H	H⋯*A*	*D*⋯*A*	*D*—H⋯*A*
N1—H1*A*⋯O2^i^	0.90 (3)	1.95 (3)	2.840 (2)	171 (2)
N2—H2*A*⋯S1^ii^	0.90 (2)	2.61 (2)	3.506 (1)	177 (2)
N3—H3*A*⋯O1^iii^	0.89 (2)	1.99 (2)	2.867 (2)	166 (2)
N4—H4*A*⋯S2^iv^	0.89 (2)	2.63 (2)	3.511 (1)	176 (2)
C3—H3⋯O1^v^	0.93	2.53	3.418 (2)	161

Symmetry codes: (i) 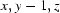; (ii) 
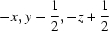
; (iii) 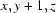; (iv) 
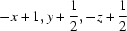
; (v) 
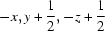
.

**Fig. 1.**
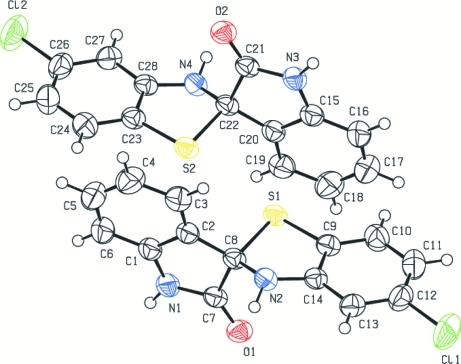
The title compound (3), with the atom-numbering scheme, intramolecular H-bonds and 50% probability displacement ellipsoids.

**Fig. 2.**
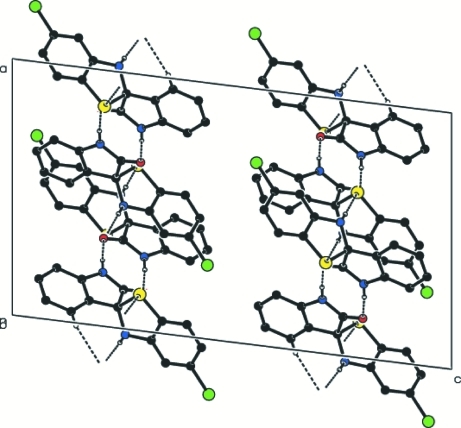
The packing and hydrogen bonding of the title compound (3) down the b axis. H atoms not involved in hydrogen bonding have been omitted.

## supplementary crystallographic information

### Comment

1*H*-Indole-2,3-dione is a synthetically versatile molecule which has led to an array of derivatives displaying a broad spectrum of biological properties including anticancer, antiviral, antituberculosis and antibacterial activities (Vine *et al.*, 2007, 2009; Quenelle *et al.*, 2006). Investigation of the structure-activity relationships in 2-indolinones revealed that cyclization to thiazolines and spiroindolinones are associated with increased activity against a range of human cancer cell lines, various bacteria and viruses (Hall *et al.*, 2009; Kumar *et al.*, 2008). A large number of 2-arylbenzothiazoles have been prepared because of their wide pharmacological potential. This important class of compounds has significant anticancer and antimicrobial properties (Ćaleta *et al.*,2009; Cho *et al.*, 2008). The reactivity of 1*H*-indole-2,3-dione towards 2-aminothiophenol has been the subject of a number of reports and some of the products obtained are quite interesting. The first results reported that 1*H*-indole-2,3-dione furnished benzothiazinone, indolobenzothiazide and spiro benzothiazole when the reaction was carried out in dry xylene in the presence of anhydrous zinc chloride under reflux. On the other hand, the reaction of 1-methyl-1*H*-indole-2,3-dione with 2-amino thiophenol under the same conditions furnished solely the spiro compound (Joshi *et al.*, 1990; Dandia *et al.*, 1990; Da-Silva *et al.*, 2001). In addition, there is one report on the reaction of 1*H*-indole-2,3-dione with 2-aminothiophenol in ethanol yielding a single spirobenzothiazole (Alam & Nawwar, 2002). Promoted by the above observations and in continuation of our study on the indolinone derivatives, we synthesized the title compound (3) by incorporating the benzothiazole moiety. Thus spectroscopic and X-ray diffraction studies were carried out on (3) to determine the spiro benzothiazole structure.

Fig. 1 shows the two crystallographically independent molecules in the asymmetric unit. Bond lengths in both molecules are within normal ranges (Allen *et al.*, 1987). The five-membered rings S1/N2/C8/C9/C14 and S2/N4/C22/C23/C28 of the benzothiazole groups in both molecules A and B [A: S1/Cl1/O1/N1/N2/C1–C14 and B: S2/Cl2/O2/N3/N4/C15–C28] adopt an envelope conformation with atom C8 at the flap for molecule A [puckering parameters are *q*_2_ = 0.242 (1) Å and φ_2_= 217.5 (4)° (Cremer & Pople, 1975)] and with atom C22 at the flap for molecule B [puckering parameters are *q*_2_ = 0.234 (1) Å and φ_2_= 37.7 (4)°]. The five-membered rings N1/C1/C2/C7/C8 and N3/C15/C20—C22 of the indolinone groups in both molecules A and B also are not planar, with twisted C7—C8 and C21—C22 bonds, respectively, [puckering parameters are q_2_ = 0.112 (2) Å and φ_2_ = 126.3 (8)° for A, and q_2_ = 0.108 (2) Å and φ_2_ = 306.4 (9)° for B]..

The torsion angles N1—C7—C8—N2, C2–C8–N2–C14 in A and N3—C21—C22—N4, C20—C22—N4—C28 in B are 141.0 (1)°, 148.5 (1)° and -140.3 (1) °, -147.8 (1)°, respectivley. Thus, they adopt +anti-clinal (+*ac*) and -anti-clinal (-*ac*) conformations, for molecules A and B, repectively.

The crystal packing is stabilized by intermolecular N—H···O, N—H···S and C—H···O hydrogen bonding interactions, forming the layers of molecules which are paralel to the (001) planes (Table 1 and Fig. 2).

### Experimental

To a solution of 1*H*-indole-2,3-dione 1 (3.5 mmol) in absolute ethanol (15 ml) was added 2-aminothiophenol 2 (3.5 mmol). The mixture was heated under reflux for 5 h. The solid thus obtained (3) was filtered, dried and recrystallized from ethanol (Alam & Nawwar, 2002). Yield: 86%; m.p.: 514 K; IR (KBr) ν (cm^-1^): 3281, 3149 (N—H), 1728(C=O); ^1^H-NMR (DMSO-d_6_, 500 MHz) δ (p.p.m.): 6.50 (1*H*, d, J = 0.96 Hz, C_13_—H), 6.62 (1*H*, dd, J = 8.30, 2.44 Hz, C_11_—H), 6.84 (1*H*, d, J = 7.81 Hz, C_6_—H), 7.03 (1*H*, d, J = 8.30 Hz, C_10_—H), 7.05 (1*H*, dt, J =7.81 Hz, C_4_—H), 7.29 (1*H*, dt, J = 7.81 Hz, C_5_—H), 7.55 (1*H*, d, J = 2.92 Hz, C_3_—H), 7.56 (1*H*, s, N_2_—H), 10.39 (1*H*, s, N_1_—H); ^13^C-NMR (HSQC) (125 MHz)(DMSO-d_6_/TMS) δ (p.p.m.): 75.64 (C_8_), 108.40 (C_13_), 118.52 (C_11_), 110.88 (C_6_), 122.70 (C_10_), 123.28 (C_4_), 123.99 (C_2_), 126.38 (C_3_), 129.86(C_9_), 130.92 (C_12_), 131.43 (C_5_), 142.03 (C_1_), 149.43 (C_14_), 176.53 (C_7_). MS (ESI+) m/*z* (%): 289 (MH^+^, 35), 287 (100). Analysis calculated for C_14_H_9_ClN_2_OS: C 58.23, H 3.14, N 9.70%. Found: C 58.06, H 3.14, N 9.52%.

### Refinement

H atoms bound to N atoms were located from a difference Fourier map and refined freely. H atoms bound to C atoms were positioned geometrically with C—H = 0.93 Å and refined using a riding model with *U*_iso_(H) = 1.2*U*_eq_(C).

### Crystal data

**Table:**

C_14_H_9_ClN_2_OS	*F*(000) = 1184
*M**_r_* = 288.75	*D*_x_ = 1.490 Mg m^−^^3^
Monoclinic, *P*2_1_/*c*	Mo *K*α radiation, λ = 0.71073 Å
Hall symbol: -P 2ybc	Cell parameters from 33214 reflections
*a* = 12.8421 (6) Å	θ = 1.6–28.0°
*b* = 9.1159 (3) Å	µ = 0.45 mm^−^^1^
*c* = 22.1553 (9) Å	*T* = 295 K
β = 97.051 (3)°	Prism, yellow
*V* = 2574.1 (2) Å^3^	0.77 × 0.49 × 0.19 mm
*Z* = 8	

### Data collection

**Table:**

Stoe IPDS 2 diffractometer	5271 independent reflections
Radiation source: sealed X-ray tube, 12 x 0.4 mm long-fine focus	3987 reflections with *I* > 2σ(*I*)
plane graphite	*R*_int_ = 0.031
Detector resolution: 6.67 pixels mm^-1^	θ_max_ = 26.5°, θ_min_ = 1.9°
ω scans	*h* = −14→16
Absorption correction: integration (*X-RED32*; Stoe & Cie, 2002)	*k* = −11→11
*T*_min_ = 0.723, *T*_max_ = 0.919	*l* = −27→27
23976 measured reflections	

### Refinement

**Table:**

Refinement on *F*^2^	Primary atom site location: structure-invariant direct methods
Least-squares matrix: full	Secondary atom site location: difference Fourier map
*R*[*F*^2^ > 2σ(*F*^2^)] = 0.033	Hydrogen site location: inferred from neighbouring sites
*wR*(*F*^2^) = 0.080	H atoms treated by a mixture of independent and constrained refinement
*S* = 1.01	*w* = 1/[σ^2^(*F*_o_^2^) + (0.0445*P*)^2^ + 0.0864*P*] where *P* = (*F*_o_^2^ + 2*F*_c_^2^)/3
5271 reflections	(Δ/σ)_max_ < 0.001
359 parameters	Δρ_max_ = 0.17 e Å^−^^3^
0 restraints	Δρ_min_ = −0.19 e Å^−^^3^

### Special details

**Table:**

Geometry. Bond distances, angles *etc*. have been calculated using the rounded fractional coordinates. All su's are estimated from the variances of the (full) variance-covariance matrix. The cell e.s.d.'s are taken into account in the estimation of distances, angles and torsion angles
Refinement. Refinement on *F*^2^ for ALL reflections except those flagged by the user for potential systematic errors. Weighted *R*-factors *wR* and all goodnesses of fit *S* are based on *F*^2^, conventional *R*-factors *R* are based on *F*, with *F* set to zero for negative *F*^2^. The observed criterion of *F*^2^ > σ(*F*^2^) is used only for calculating -*R*-factor-obs *etc*. and is not relevant to the choice of reflections for refinement. *R*-factors based on *F*^2^ are statistically about twice as large as those based on *F*, and *R*-factors based on ALL data will be even larger.

### Fractional atomic coordinates and isotropic or equivalent isotropic displacement parameters (Å^2^)

**Table:**

	*x*	*y*	*z*	*U*_iso_*/*U*_eq_	
Cl1	−0.21534 (5)	0.20753 (8)	0.44750 (3)	0.0897 (3)	
S1	0.14151 (3)	0.34132 (4)	0.29015 (2)	0.0398 (1)	
O1	0.15538 (9)	−0.02243 (11)	0.29802 (5)	0.0468 (4)	
N1	0.20968 (12)	0.04018 (14)	0.20602 (7)	0.0459 (5)	
N2	−0.02266 (11)	0.17509 (14)	0.25719 (6)	0.0386 (4)	
C1	0.17319 (13)	0.13376 (17)	0.15826 (7)	0.0439 (5)	
C2	0.09339 (13)	0.22368 (16)	0.17481 (7)	0.0407 (5)	
C3	0.04229 (16)	0.32123 (18)	0.13402 (8)	0.0528 (6)	
C4	0.0736 (2)	0.3287 (2)	0.07608 (9)	0.0684 (8)	
C5	0.1542 (2)	0.2413 (2)	0.06049 (9)	0.0682 (8)	
C6	0.20529 (17)	0.1426 (2)	0.10113 (9)	0.0581 (7)	
C7	0.15275 (12)	0.05484 (15)	0.25322 (7)	0.0384 (5)	
C8	0.08181 (12)	0.19188 (15)	0.24007 (7)	0.0371 (4)	
C9	0.04814 (13)	0.31467 (15)	0.34106 (7)	0.0400 (5)	
C10	0.04669 (15)	0.3771 (2)	0.39753 (8)	0.0538 (6)	
C11	−0.03608 (18)	0.3447 (2)	0.43046 (9)	0.0603 (7)	
C12	−0.11329 (15)	0.2499 (2)	0.40578 (8)	0.0541 (6)	
C13	−0.11335 (13)	0.18672 (17)	0.34914 (8)	0.0446 (5)	
C14	−0.03182 (12)	0.22070 (15)	0.31617 (7)	0.0366 (4)	
Cl2	0.71172 (5)	0.55718 (7)	0.05591 (3)	0.0844 (2)	
S2	0.35504 (3)	0.42746 (4)	0.21381 (2)	0.0400 (1)	
O2	0.34292 (10)	0.79114 (12)	0.20783 (5)	0.0470 (4)	
N3	0.29057 (12)	0.72689 (14)	0.30029 (7)	0.0456 (4)	
N4	0.52018 (11)	0.59219 (14)	0.24673 (6)	0.0388 (4)	
C15	0.32736 (13)	0.63104 (16)	0.34761 (8)	0.0441 (5)	
C16	0.29684 (17)	0.6217 (2)	0.40483 (9)	0.0589 (7)	
C17	0.3485 (2)	0.5204 (2)	0.44442 (9)	0.0689 (8)	
C18	0.4273 (2)	0.4313 (2)	0.42740 (9)	0.0665 (8)	
C19	0.45735 (15)	0.44084 (18)	0.36933 (8)	0.0505 (6)	
C20	0.40570 (13)	0.54115 (16)	0.32944 (7)	0.0406 (5)	
C21	0.34576 (12)	0.71277 (15)	0.25252 (7)	0.0381 (5)	
C22	0.41620 (12)	0.57500 (14)	0.26426 (7)	0.0363 (4)	
C23	0.44832 (13)	0.45380 (15)	0.16277 (7)	0.0405 (5)	
C24	0.44918 (16)	0.3916 (2)	0.10620 (8)	0.0535 (6)	
C25	0.53209 (18)	0.4225 (2)	0.07331 (9)	0.0616 (7)	
C26	0.60934 (15)	0.5167 (2)	0.09761 (8)	0.0535 (6)	
C27	0.60988 (13)	0.58121 (17)	0.15439 (8)	0.0443 (5)	
C28	0.52871 (12)	0.54685 (15)	0.18761 (7)	0.0368 (4)	
H1A	0.257 (2)	−0.032 (3)	0.2053 (10)	0.081 (7)*	
H2A	−0.0558 (16)	0.091 (2)	0.2451 (8)	0.056 (5)*	
H3	−0.01150	0.38030	0.14480	0.0630*	
H4	0.04000	0.39320	0.04750	0.0820*	
H5	0.17450	0.24920	0.02170	0.0820*	
H6	0.25940	0.08410	0.09040	0.0700*	
H10	0.10020	0.43990	0.41350	0.0650*	
H11	−0.03920	0.38640	0.46850	0.0720*	
H13	−0.16670	0.12320	0.33360	0.0530*	
H3A	0.2402 (19)	0.794 (2)	0.3016 (9)	0.070 (6)*	
H4A	0.5540 (16)	0.674 (2)	0.2583 (8)	0.050 (5)*	
H16	0.24380	0.68090	0.41650	0.0710*	
H17	0.32980	0.51190	0.48350	0.0830*	
H18	0.46040	0.36450	0.45510	0.0800*	
H19	0.51040	0.38170	0.35770	0.0610*	
H24	0.39510	0.32980	0.09020	0.0640*	
H25	0.53510	0.37990	0.03540	0.0740*	
H27	0.66300	0.64540	0.16960	0.0530*	

### Atomic displacement parameters (Å^2^)

**Table:**

	*U*^11^	*U*^22^	*U*^33^	*U*^12^	*U*^13^	*U*^23^
Cl1	0.0863 (5)	0.1113 (5)	0.0817 (4)	−0.0106 (4)	0.0518 (4)	0.0006 (3)
S1	0.0346 (2)	0.0328 (2)	0.0528 (2)	−0.0029 (2)	0.0087 (2)	−0.0030 (2)
O1	0.0451 (7)	0.0386 (5)	0.0585 (7)	0.0065 (5)	0.0138 (6)	0.0070 (5)
N1	0.0440 (8)	0.0377 (7)	0.0591 (9)	0.0076 (6)	0.0185 (7)	−0.0010 (6)
N2	0.0303 (7)	0.0369 (6)	0.0492 (8)	−0.0010 (5)	0.0073 (6)	−0.0045 (6)
C1	0.0438 (9)	0.0372 (7)	0.0527 (9)	−0.0045 (7)	0.0143 (7)	−0.0059 (7)
C2	0.0425 (9)	0.0345 (7)	0.0462 (9)	−0.0026 (6)	0.0095 (7)	−0.0031 (6)
C3	0.0586 (12)	0.0463 (9)	0.0536 (10)	0.0033 (8)	0.0075 (9)	0.0017 (7)
C4	0.0886 (18)	0.0634 (12)	0.0530 (11)	−0.0042 (11)	0.0074 (11)	0.0111 (9)
C5	0.0888 (17)	0.0686 (12)	0.0511 (11)	−0.0163 (12)	0.0241 (11)	−0.0036 (9)
C6	0.0643 (13)	0.0544 (10)	0.0604 (11)	−0.0060 (9)	0.0272 (10)	−0.0081 (9)
C7	0.0326 (8)	0.0307 (7)	0.0530 (9)	−0.0008 (6)	0.0092 (7)	−0.0022 (6)
C8	0.0336 (8)	0.0315 (7)	0.0468 (8)	0.0016 (6)	0.0080 (7)	−0.0016 (6)
C9	0.0371 (9)	0.0345 (7)	0.0487 (9)	0.0003 (6)	0.0060 (7)	0.0005 (6)
C10	0.0572 (12)	0.0538 (9)	0.0505 (10)	−0.0064 (9)	0.0072 (8)	−0.0098 (8)
C11	0.0680 (14)	0.0663 (12)	0.0488 (10)	0.0010 (10)	0.0163 (9)	−0.0085 (9)
C12	0.0537 (12)	0.0582 (10)	0.0543 (10)	0.0052 (9)	0.0225 (9)	0.0079 (8)
C13	0.0369 (9)	0.0421 (8)	0.0561 (10)	0.0020 (7)	0.0109 (8)	0.0049 (7)
C14	0.0320 (8)	0.0329 (7)	0.0453 (8)	0.0044 (6)	0.0063 (6)	0.0016 (6)
Cl2	0.0786 (4)	0.1071 (4)	0.0763 (4)	−0.0002 (3)	0.0450 (3)	0.0068 (3)
S2	0.0353 (2)	0.0323 (2)	0.0531 (2)	−0.0031 (2)	0.0084 (2)	−0.0037 (2)
O2	0.0452 (7)	0.0385 (6)	0.0590 (7)	0.0070 (5)	0.0134 (6)	0.0055 (5)
N3	0.0422 (8)	0.0373 (6)	0.0605 (9)	0.0076 (6)	0.0194 (7)	−0.0010 (6)
N4	0.0308 (7)	0.0364 (6)	0.0499 (8)	−0.0023 (5)	0.0081 (6)	−0.0070 (6)
C15	0.0448 (10)	0.0357 (7)	0.0535 (9)	−0.0046 (7)	0.0133 (7)	−0.0041 (7)
C16	0.0678 (13)	0.0531 (10)	0.0604 (11)	−0.0056 (9)	0.0269 (10)	−0.0083 (9)
C17	0.0906 (17)	0.0673 (12)	0.0523 (11)	−0.0088 (12)	0.0234 (11)	0.0001 (9)
C18	0.0848 (17)	0.0620 (11)	0.0517 (11)	0.0016 (11)	0.0048 (10)	0.0102 (9)
C19	0.0544 (11)	0.0429 (8)	0.0534 (10)	0.0025 (7)	0.0037 (8)	0.0004 (7)
C20	0.0404 (9)	0.0350 (7)	0.0470 (9)	−0.0031 (6)	0.0084 (7)	−0.0042 (6)
C21	0.0313 (8)	0.0311 (7)	0.0526 (9)	−0.0008 (6)	0.0080 (7)	−0.0029 (6)
C22	0.0319 (8)	0.0294 (7)	0.0481 (8)	0.0012 (6)	0.0069 (7)	−0.0025 (6)
C23	0.0382 (9)	0.0343 (7)	0.0495 (9)	0.0023 (6)	0.0075 (7)	−0.0002 (6)
C24	0.0593 (12)	0.0509 (9)	0.0502 (9)	−0.0062 (8)	0.0062 (8)	−0.0075 (8)
C25	0.0736 (15)	0.0652 (12)	0.0482 (10)	0.0052 (10)	0.0169 (10)	−0.0062 (9)
C26	0.0538 (11)	0.0563 (10)	0.0538 (10)	0.0076 (8)	0.0204 (9)	0.0078 (8)
C27	0.0360 (9)	0.0429 (8)	0.0551 (10)	0.0027 (7)	0.0102 (7)	0.0051 (7)
C28	0.0324 (8)	0.0312 (7)	0.0469 (8)	0.0044 (6)	0.0054 (6)	0.0013 (6)

### Geometric parameters (Å, °)

**Table:**

Cl1—C12	1.737 (2)	C11—C12	1.377 (3)
Cl2—C26	1.737 (2)	C12—C13	1.381 (2)
S1—C9	1.7609 (17)	C13—C14	1.383 (2)
S1—C8	1.8617 (15)	C3—H3	0.9300
S2—C22	1.8600 (15)	C4—H4	0.9300
S2—C23	1.7619 (17)	C5—H5	0.9300
O1—C7	1.2142 (18)	C6—H6	0.9300
O2—C21	1.2179 (18)	C10—H10	0.9300
N1—C7	1.354 (2)	C11—H11	0.9300
N1—C1	1.395 (2)	C13—H13	0.9300
N2—C14	1.390 (2)	C15—C16	1.375 (3)
N2—C8	1.446 (2)	C15—C20	1.395 (2)
N1—H1A	0.90 (3)	C16—C17	1.385 (3)
N2—H2A	0.901 (19)	C17—C18	1.386 (3)
N3—C15	1.402 (2)	C18—C19	1.391 (3)
N3—C21	1.350 (2)	C19—C20	1.383 (2)
N4—C28	1.391 (2)	C20—C22	1.499 (2)
N4—C22	1.444 (2)	C21—C22	1.551 (2)
N3—H3A	0.89 (2)	C23—C28	1.396 (2)
N4—H4A	0.885 (19)	C23—C24	1.377 (2)
C1—C2	1.396 (2)	C24—C25	1.391 (3)
C1—C6	1.381 (3)	C25—C26	1.371 (3)
C2—C8	1.500 (2)	C26—C27	1.388 (2)
C2—C3	1.376 (2)	C27—C28	1.384 (2)
C3—C4	1.393 (3)	C16—H16	0.9300
C4—C5	1.383 (3)	C17—H17	0.9300
C5—C6	1.381 (3)	C18—H18	0.9300
C7—C8	1.553 (2)	C19—H19	0.9300
C9—C14	1.398 (2)	C24—H24	0.9300
C9—C10	1.377 (2)	C25—H25	0.9300
C10—C11	1.393 (3)	C27—H27	0.9300
			
Cl1···Cl2^i^	3.6085 (10)	C13···O2^vii^	3.206 (2)
Cl2···Cl1^ii^	3.6085 (10)	C13···C21^vii^	3.520 (2)
Cl2···H5^iii^	2.9700	C13···C3^vii^	3.463 (2)
S1···O1	3.3241 (11)	C14···O1	3.3303 (18)
S1···N1	3.4896 (14)	C15···S1	3.6805 (16)
S1···N2^iv^	3.5063 (14)	C15···C25^ix^	3.551 (3)
S1···C15	3.6805 (16)	C16···C25^ix^	3.510 (3)
S1···S2	3.4836 (6)	C19···C27^v^	3.414 (2)
S2···N4^v^	3.5113 (14)	C21···C13^iv^	3.520 (2)
S2···S1	3.4836 (6)	C25···C16^v^	3.510 (3)
S2···O2	3.3208 (12)	C25···C15^v^	3.551 (3)
S2···N3	3.4923 (14)	C25···C25^iii^	3.546 (3)
S2···C1	3.6649 (16)	C27···C19^ix^	3.414 (2)
S1···H2A^iv^	2.606 (19)	C27···C7^ix^	3.465 (2)
S1···H27^v^	3.1200	C27···O1^ix^	3.211 (2)
S2···H13^iv^	3.0800	C28···O2	3.3339 (19)
S2···H4A^v^	2.628 (19)	C6···H24	3.0100
O1···N2	2.9632 (18)	C7···H3A^vi^	2.787 (19)
O1···N3^vi^	2.8666 (18)	C7···H27^v^	2.8700
O1···S1	3.3241 (11)	C16···H10	3.0500
O1···C3^vii^	3.418 (2)	C21···H1A^viii^	2.74 (3)
O1···C14	3.3303 (18)	C21···H13^iv^	2.9200
O1···C27^v^	3.211 (2)	C23···H4A^v^	3.095 (18)
O2···C28	3.3339 (19)	C25···H25^iii^	3.0500
O2···N1^viii^	2.8402 (18)	H1A···C21^vi^	2.74 (3)
O2···N4	2.9550 (18)	H1A···O2^vi^	1.95 (3)
O2···S2	3.3208 (12)	H2A···S1^vii^	2.606 (19)
O2···C13^iv^	3.206 (2)	H2A···H13	2.5800
O1···H3A^vi^	1.99 (2)	H3···O1^iv^	2.5300
O1···H27^v^	2.8100	H3A···C7^viii^	2.787 (19)
O1···H3^vii^	2.5300	H3A···O1^viii^	1.99 (2)
O2···H1A^viii^	1.95 (3)	H4A···S2^ix^	2.628 (19)
O2···H13^iv^	2.7900	H4A···C23^ix^	3.095 (18)
O2···H19^ix^	2.6500	H4A···H27	2.5600
N1···S1	3.4896 (14)	H5···Cl2^iii^	2.9700
N1···O2^vi^	2.8402 (18)	H10···C16	3.0500
N2···S1^vii^	3.5063 (14)	H13···O2^vii^	2.7900
N2···O1	2.9632 (18)	H13···C21^vii^	2.9200
N3···O1^viii^	2.8666 (18)	H13···S2^vii^	3.0800
N3···S2	3.4923 (14)	H13···H2A	2.5800
N4···O2	2.9550 (18)	H19···O2^v^	2.6500
N4···S2^ix^	3.5113 (14)	H24···C6	3.0100
C1···S2	3.6649 (16)	H25···C25^iii^	3.0500
C3···C13^iv^	3.463 (2)	H27···H4A	2.5600
C3···O1^iv^	3.418 (2)	H27···S1^ix^	3.1200
C6···C11^vii^	3.495 (3)	H27···O1^ix^	2.8100
C7···C27^v^	3.465 (2)	H27···C7^ix^	2.8700
C11···C6^iv^	3.495 (3)		
			
C8—S1—C9	91.02 (7)	C5—C6—H6	121.00
C22—S2—C23	90.97 (7)	C9—C10—H10	121.00
C1—N1—C7	111.26 (14)	C11—C10—H10	120.00
C8—N2—C14	113.78 (13)	C10—C11—H11	121.00
C7—N1—H1A	121.2 (15)	C12—C11—H11	120.00
C1—N1—H1A	126.9 (14)	C12—C13—H13	121.00
C8—N2—H2A	115.6 (13)	C14—C13—H13	121.00
C14—N2—H2A	116.3 (12)	C16—C15—C20	121.82 (16)
C15—N3—C21	111.45 (14)	N3—C15—C16	128.34 (16)
C22—N4—C28	113.79 (13)	N3—C15—C20	109.83 (15)
C21—N3—H3A	122.9 (13)	C15—C16—C17	117.16 (19)
C15—N3—H3A	125.6 (13)	C16—C17—C18	121.83 (19)
C28—N4—H4A	115.5 (12)	C17—C18—C19	120.67 (18)
C22—N4—H4A	116.8 (13)	C18—C19—C20	117.82 (17)
N1—C1—C2	110.43 (14)	C15—C20—C19	120.69 (15)
N1—C1—C6	128.38 (16)	C15—C20—C22	108.09 (13)
C2—C1—C6	121.19 (15)	C19—C20—C22	131.22 (15)
C3—C2—C8	131.49 (15)	O2—C21—N3	127.96 (14)
C1—C2—C8	107.61 (13)	O2—C21—C22	124.75 (14)
C1—C2—C3	120.89 (15)	N3—C21—C22	107.29 (12)
C2—C3—C4	117.94 (18)	S2—C22—C20	110.41 (10)
C3—C4—C5	120.76 (18)	S2—C22—C21	106.87 (10)
C4—C5—C6	121.53 (19)	S2—C22—N4	104.66 (10)
C1—C6—C5	117.68 (19)	C20—C22—C21	102.05 (12)
O1—C7—C8	125.24 (14)	N4—C22—C20	118.49 (13)
O1—C7—N1	127.63 (14)	N4—C22—C21	113.99 (12)
N1—C7—C8	107.13 (12)	S2—C23—C28	110.98 (11)
S1—C8—C2	110.54 (10)	C24—C23—C28	121.39 (16)
N2—C8—C2	118.57 (13)	S2—C23—C24	127.60 (13)
N2—C8—C7	114.05 (12)	C23—C24—C25	119.06 (17)
S1—C8—N2	104.39 (10)	C24—C25—C26	118.99 (18)
C2—C8—C7	102.19 (12)	Cl2—C26—C25	118.86 (15)
S1—C8—C7	106.72 (10)	Cl2—C26—C27	118.18 (14)
S1—C9—C14	110.91 (11)	C25—C26—C27	122.96 (18)
C10—C9—C14	121.38 (16)	C26—C27—C28	117.77 (15)
S1—C9—C10	127.68 (13)	N4—C28—C27	125.83 (14)
C9—C10—C11	119.00 (17)	C23—C28—C27	119.79 (14)
C10—C11—C12	118.93 (18)	N4—C28—C23	114.33 (14)
C11—C12—C13	122.87 (18)	C15—C16—H16	121.00
Cl1—C12—C11	118.80 (15)	C17—C16—H16	121.00
Cl1—C12—C13	118.33 (14)	C16—C17—H17	119.00
C12—C13—C14	118.07 (15)	C18—C17—H17	119.00
N2—C14—C13	125.90 (14)	C17—C18—H18	120.00
C9—C14—C13	119.73 (14)	C19—C18—H18	120.00
N2—C14—C9	114.29 (14)	C18—C19—H19	121.00
C4—C3—H3	121.00	C20—C19—H19	121.00
C2—C3—H3	121.00	C23—C24—H24	120.00
C3—C4—H4	120.00	C25—C24—H24	120.00
C5—C4—H4	120.00	C24—C25—H25	121.00
C6—C5—H5	119.00	C26—C25—H25	121.00
C4—C5—H5	119.00	C26—C27—H27	121.00
C1—C6—H6	121.00	C28—C27—H27	121.00
			
C9—S1—C8—C7	−101.67 (11)	O1—C7—C8—S1	75.43 (17)
C8—S1—C9—C10	171.57 (16)	C10—C9—C14—N2	175.71 (15)
C8—S1—C9—C14	−10.32 (12)	C14—C9—C10—C11	0.4 (3)
C9—S1—C8—N2	19.41 (10)	C10—C9—C14—C13	−1.3 (2)
C9—S1—C8—C2	147.97 (11)	S1—C9—C14—C13	−179.58 (12)
C23—S2—C22—C21	102.41 (11)	S1—C9—C10—C11	178.36 (14)
C23—S2—C22—C20	−147.38 (11)	S1—C9—C14—N2	−2.55 (16)
C22—S2—C23—C28	9.92 (12)	C9—C10—C11—C12	0.7 (3)
C23—S2—C22—N4	−18.82 (10)	C10—C11—C12—Cl1	179.01 (15)
C22—S2—C23—C24	−172.01 (16)	C10—C11—C12—C13	−1.0 (3)
C1—N1—C7—O1	169.93 (15)	C11—C12—C13—C14	0.1 (3)
C1—N1—C7—C8	−10.37 (17)	Cl1—C12—C13—C14	−179.91 (13)
C7—N1—C1—C6	−174.55 (17)	C12—C13—C14—C9	1.0 (2)
C7—N1—C1—C2	4.51 (19)	C12—C13—C14—N2	−175.62 (15)
C8—N2—C14—C9	19.25 (18)	C16—C15—C20—C19	−1.5 (3)
C8—N2—C14—C13	−163.92 (14)	C16—C15—C20—C22	177.60 (16)
C14—N2—C8—S1	−25.03 (14)	N3—C15—C20—C19	177.41 (15)
C14—N2—C8—C2	−148.54 (13)	N3—C15—C20—C22	−3.54 (18)
C14—N2—C8—C7	91.06 (15)	C20—C15—C16—C17	1.0 (3)
C15—N3—C21—C22	10.02 (17)	N3—C15—C16—C17	−177.60 (18)
C21—N3—C15—C16	174.40 (17)	C15—C16—C17—C18	−0.3 (3)
C15—N3—C21—O2	−169.98 (16)	C16—C17—C18—C19	0.0 (3)
C21—N3—C15—C20	−4.37 (19)	C17—C18—C19—C20	−0.3 (3)
C22—N4—C28—C23	−18.74 (18)	C18—C19—C20—C15	1.1 (3)
C28—N4—C22—C20	147.82 (13)	C18—C19—C20—C22	−177.75 (17)
C22—N4—C28—C27	164.02 (14)	C15—C20—C22—S2	−104.54 (13)
C28—N4—C22—C21	−92.09 (15)	C15—C20—C22—C21	8.79 (16)
C28—N4—C22—S2	24.31 (14)	C19—C20—C22—S2	74.4 (2)
N1—C1—C6—C5	177.63 (18)	C15—C20—C22—N4	134.86 (14)
N1—C1—C2—C3	−177.35 (15)	C19—C20—C22—N4	−46.2 (2)
C2—C1—C6—C5	−1.3 (3)	C19—C20—C22—C21	−172.30 (17)
C6—C1—C2—C8	−177.18 (16)	O2—C21—C22—C20	168.65 (15)
N1—C1—C2—C8	3.69 (18)	O2—C21—C22—N4	39.7 (2)
C6—C1—C2—C3	1.8 (3)	N3—C21—C22—C20	−11.35 (16)
C1—C2—C8—S1	104.15 (13)	N3—C21—C22—S2	104.59 (12)
C3—C2—C8—N2	45.7 (2)	N3—C21—C22—N4	−140.30 (13)
C3—C2—C8—C7	172.06 (17)	O2—C21—C22—S2	−75.41 (17)
C1—C2—C8—N2	−135.45 (14)	C28—C23—C24—C25	0.0 (3)
C1—C2—C8—C7	−9.13 (16)	S2—C23—C28—N4	2.57 (16)
C3—C2—C8—S1	−74.7 (2)	C24—C23—C28—N4	−175.64 (15)
C1—C2—C3—C4	−0.8 (3)	C24—C23—C28—C27	1.8 (2)
C8—C2—C3—C4	177.86 (17)	S2—C23—C28—C27	180.00 (12)
C2—C3—C4—C5	−0.5 (3)	S2—C23—C24—C25	−177.89 (14)
C3—C4—C5—C6	0.9 (3)	C23—C24—C25—C26	−1.5 (3)
C4—C5—C6—C1	0.0 (3)	C24—C25—C26—C27	1.2 (3)
N1—C7—C8—C2	11.79 (16)	C24—C25—C26—Cl2	−179.04 (15)
N1—C7—C8—N2	140.99 (13)	Cl2—C26—C27—C28	−179.19 (13)
N1—C7—C8—S1	−104.29 (12)	C25—C26—C27—C28	0.5 (3)
O1—C7—C8—C2	−168.49 (15)	C26—C27—C28—C23	−2.0 (2)
O1—C7—C8—N2	−39.3 (2)	C26—C27—C28—N4	175.09 (15)

Symmetry codes: (i) *x*−1, −*y*+1/2, *z*+1/2; (ii) *x*+1, −*y*+1/2, *z*−1/2; (iii) −*x*+1, −*y*+1, −*z*; (iv) −*x*, *y*+1/2, −*z*+1/2; (v) −*x*+1, *y*−1/2, −*z*+1/2; (vi) *x*, *y*−1, *z*; (vii) −*x*, *y*−1/2, −*z*+1/2; (viii) *x*, *y*+1, *z*; (ix) −*x*+1, *y*+1/2, −*z*+1/2.

### Hydrogen-bond geometry (Å, °)

**Table:**

*D*—H···*A*	*D*—H	H···*A*	*D*···*A*	*D*—H···*A*
N1—H1A···O2^vi^	0.90 (3)	1.95 (3)	2.840 (2)	171 (2)
N2—H2A···S1^vii^	0.90 (2)	2.61 (2)	3.506 (1)	177 (2)
N3—H3A···O1^viii^	0.89 (2)	1.99 (2)	2.867 (2)	166 (2)
N4—H4A···S2^ix^	0.89 (2)	2.63 (2)	3.511 (1)	176 (2)
C3—H3···O1^iv^	0.93	2.53	3.418 (2)	161

Symmetry codes: (vi) *x*, *y*−1, *z*; (vii) −*x*, *y*−1/2, −*z*+1/2; (viii) *x*, *y*+1, *z*; (ix) −*x*+1, *y*+1/2, −*z*+1/2; (iv) −*x*, *y*+1/2, −*z*+1/2.
